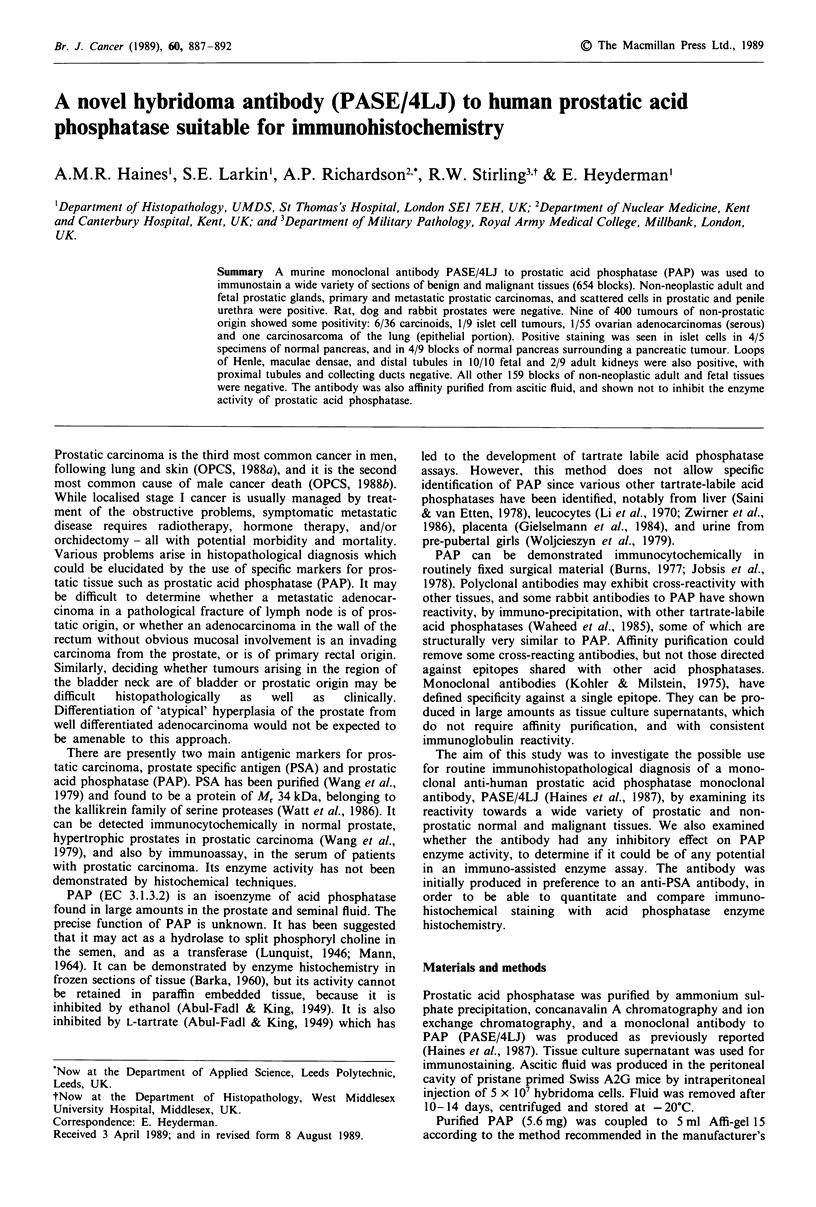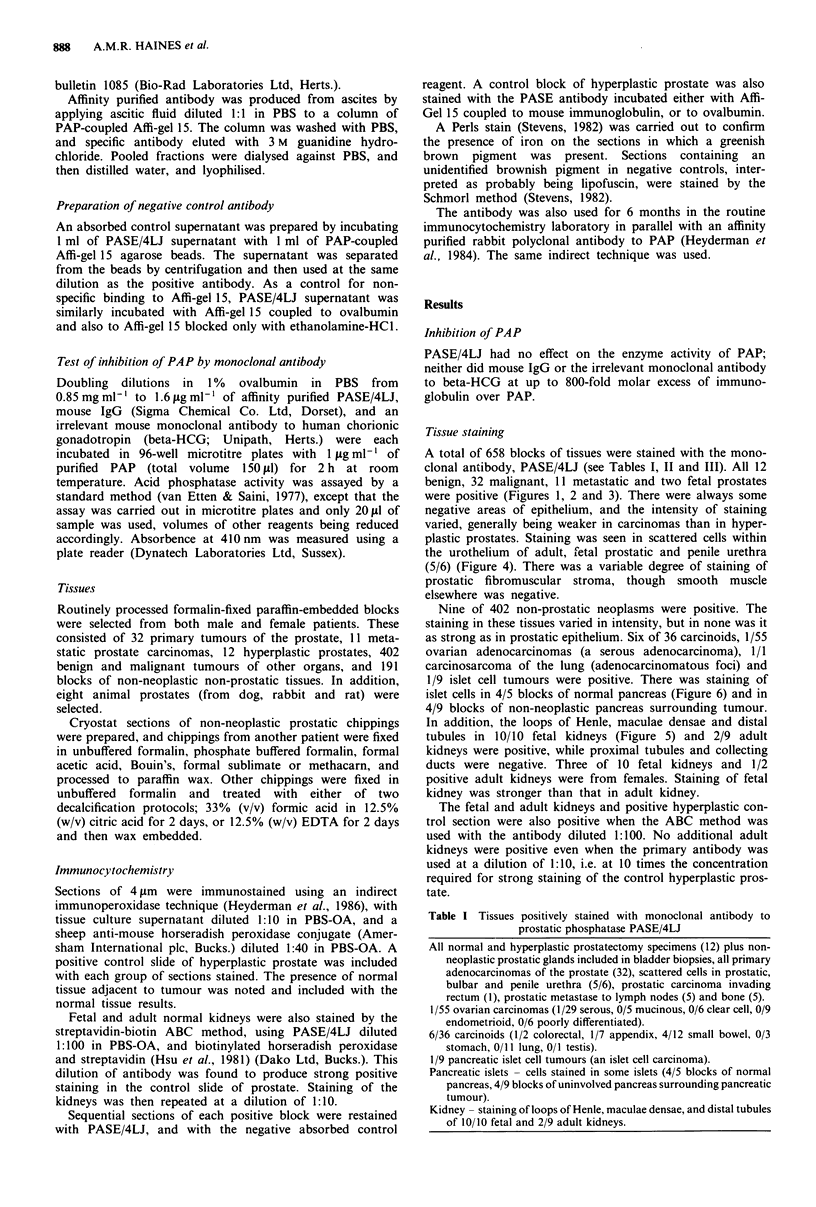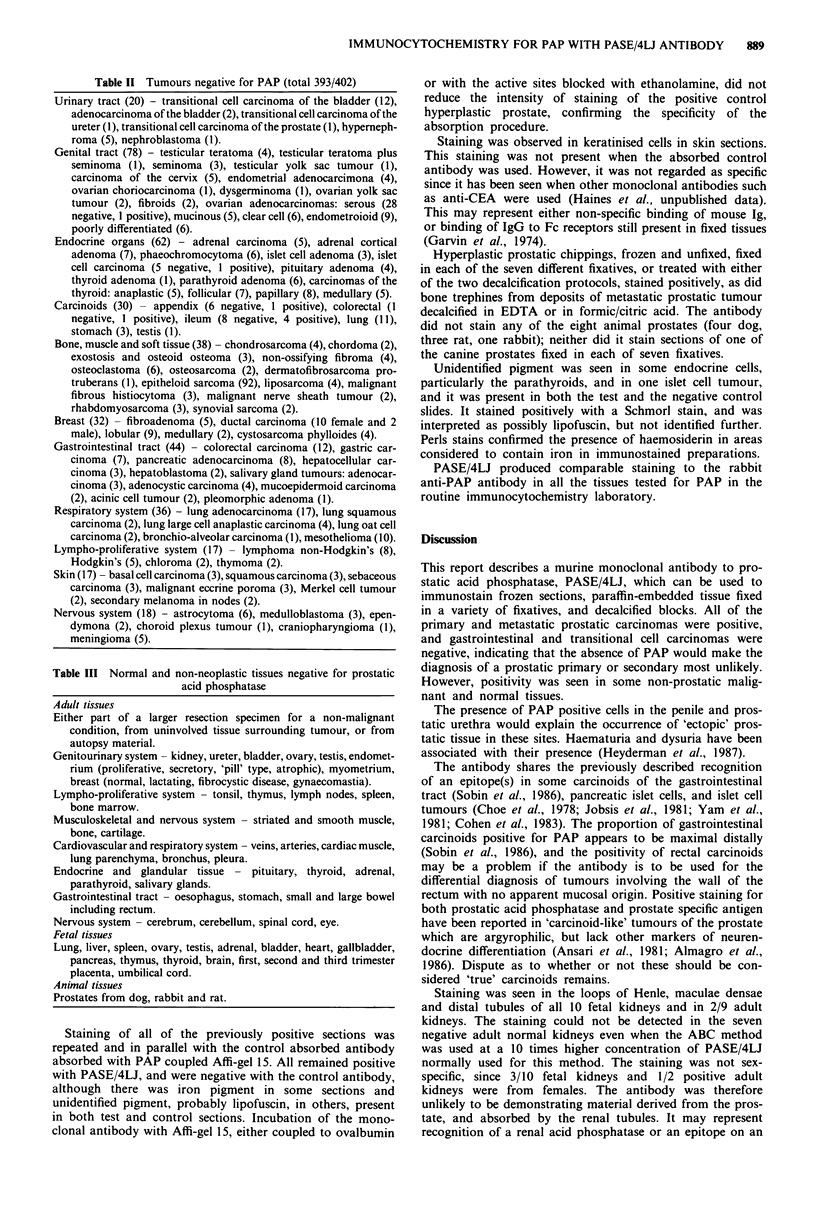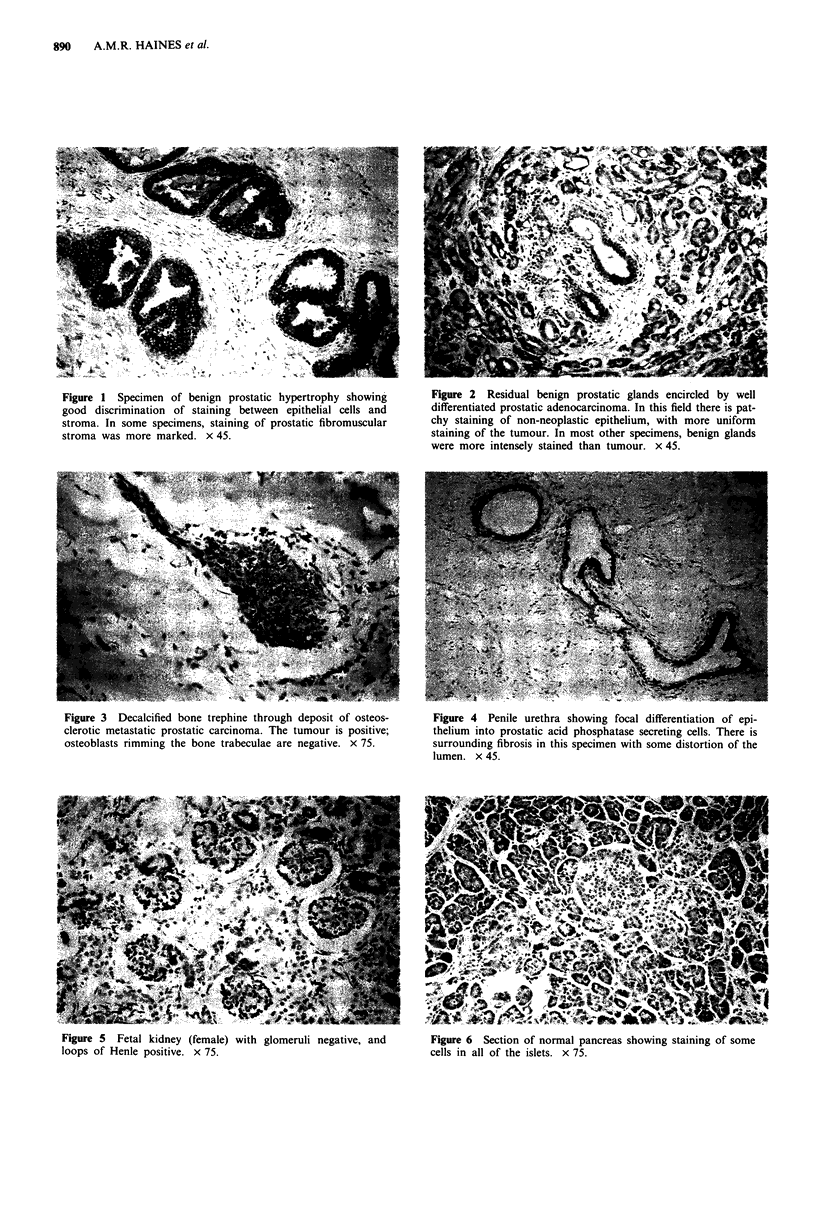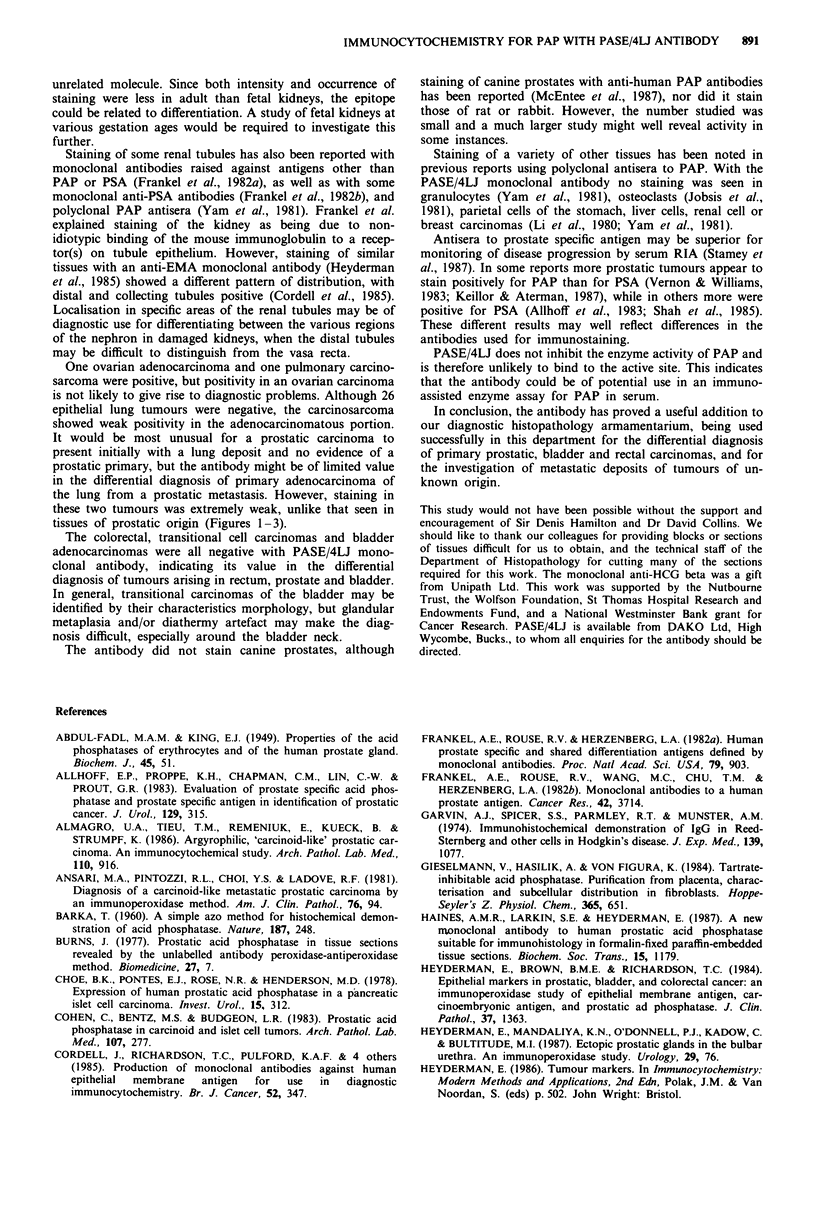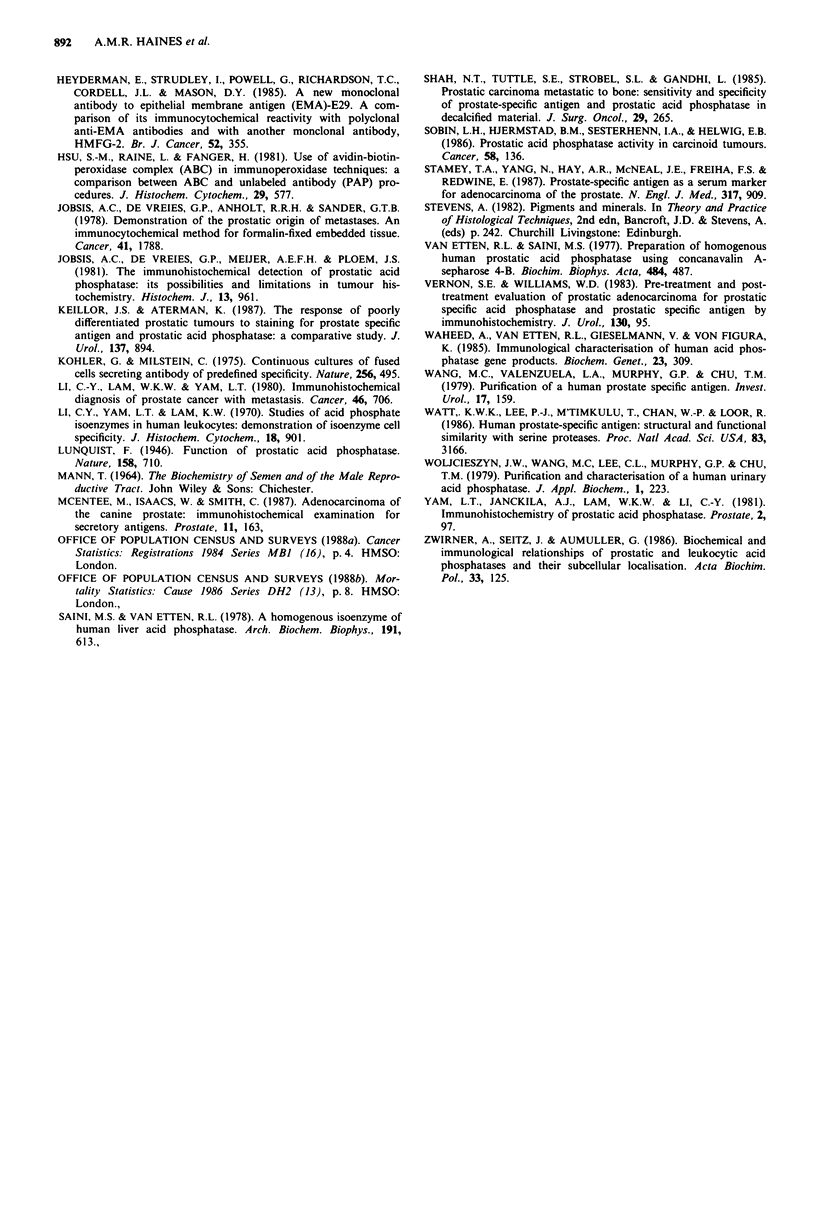# A novel hybridoma antibody (PASE/4LJ) to human prostatic acid phosphatase suitable for immunohistochemistry.

**DOI:** 10.1038/bjc.1989.385

**Published:** 1989-12

**Authors:** A. M. Haines, S. E. Larkin, A. P. Richardson, R. W. Stirling, E. Heyderman

**Affiliations:** Department of Histopathology, UMDS, St Thomas's Hospital, London, UK.

## Abstract

**Images:**


Br. J. Cancer (1989), 60, 887-892 © The Macmillan Press Ltd., 1989

### A novel hybridoma antibody (PASE/4LJ) to human prostatic acid phosphatase suitable for immunohistochemistry

A.M.R. Haines[1], S.E. Larkin[1], A.P. Richardson[2,*], R.W. Stirling[3,†] & E. Heyderman[1]

[1]*Department of Histopathology, UMDS, St Thomas's Hospital, London SE1 7EH, UK;* [2]*Department of Nuclear Medicine, Kent and Canterbury Hospital, Kent, UK; and* [3]*Department of Military Pathology, Royal Army Medical College, Millbank, London, UK.*

**Summary** A murine monoclonal antibody PASE/4LJ to prostatic acid phosphatase (PAP) was used to immunostain a wide variety of sections of benign and malignant tissues (654 blocks). Non-neoplastic adult and fetal prostatic glands, primary and metastatic prostatic carcinomas, and scattered cells in prostatic and penile urethra were positive. Rat, dog and rabbit prostates were negative. Nine of 400 tumours of non-prostatic origin showed some positivity: 6/36 carcinoids, 1/9 islet cell tumours, 1/55 ovarian adenocarcinomas (serous) and one carcinosarcoma of the lung (epithelial portion). Positive staining was seen in islet cells in 4/5 specimens of normal pancreas, and in 4/9 blocks of normal pancreas surrounding a pancreatic tumour. Loops of Henle, maculae densae, and distal tubules in 10/10 fetal and 2/9 adult kidneys were also positive, with proximal tubules and collecting ducts negative. All other 159 blocks of non-neoplastic adult and fetal tissues were negative. The antibody was also affinity purified from ascitic fluid, and shown not to inhibit the enzyme activity of prostatic acid phosphatase.

Prostatic carcinoma is the third most common cancer in men, following lung and skin (OPCS, 1988*a*), and it is the second most common cause of male cancer death (OPCS, 1988*b*). While localised stage I cancer is usually managed by treatment of the obstructive problems, symptomatic metastatic disease requires radiotherapy, hormone therapy, and/or orchidectomy – all with potential morbidity and mortality. Various problems arise in histopathological diagnosis which could be elucidated by the use of specific markers for prostatic tissue such as prostatic acid phosphatase (PAP). It may be difficult to determine whether a metastatic adenocarcinoma in a pathological fracture of lymph node is of prostatic origin, or whether an adenocarcinoma in the wall of the rectum without obvious mucosal involvement is an invading carcinoma from the prostate, or is of primary rectal origin. Similarly, deciding whether tumours arising in the region of the bladder neck are of bladder or prostatic origin may be difficult histopathologically as well as clinically. Differentiation of 'atypical' hyperplasia of the prostate from well differentiated adenocarcinoma would not be expected to be amenable to this approach.

There are presently two main antigenic markers for prostatic carcinoma, prostate specific antigen (PSA) and prostatic acid phosphatase (PAP). PSA has been purified (Wang *et al.*, 1979) and found to be a protein of $M_r$ 34 kDa, belonging to the kallikrein family of serine proteases (Watt *et al.*, 1986). It can be detected immunocytochemically in normal prostate, hypertrophic prostates in prostatic carcinoma (Wang *et al.*, 1979), and also by immunoassay, in the serum of patients with prostatic carcinoma. Its enzyme activity has not been demonstrated by histochemical techniques.

PAP (EC 3.1.3.2) is an isoenzyme of acid phosphatase found in large amounts in the prostate and seminal fluid. The precise function of PAP is unknown. It has been suggested that it may act as a hydrolase to split phosphoryl choline in the semen, and as a transferase (Lunquist, 1946; Mann, 1964). It can be demonstrated by enzyme histochemistry in frozen sections of tissue (Barka, 1960), but its activity cannot be retained in paraffin embedded tissue, because it is inhibited by ethanol (Abul-Fadl & King, 1949). It is also inhibited by L-tartrate (Abul-Fadl & King, 1949) which has led to the development of tartrate labile acid phosphatase assays. However, this method does not allow specific identification of PAP since various other tartrate-labile acid phosphatases have been identified, notably from liver (Saini & van Etten, 1978), leucocytes (Li *et al.*, 1970; Zwirner *et al.*, 1986), placenta (Gielselmann *et al.*, 1984), and urine from pre-pubertal girls (Woljcieszyn *et al.*, 1979).

PAP can be demonstrated immunocytochemically in routinely fixed surgical material (Burns, 1977; Jobsis *et al.*, 1978). Polyclonal antibodies may exhibit cross-reactivity with other tissues, and some rabbit antibodies to PAP have shown reactivity, by immuno-precipitation, with other tartrate-labile acid phosphatases (Waheed *et al.*, 1985), some of which are structurally very similar to PAP. Affinity purification could remove some cross-reacting antibodies, but not those directed against epitopes shared with other acid phosphatases. Monoclonal antibodies (Kohler & Milstein, 1975), have defined specificity against a single epitope. They can be produced in large amounts as tissue culture supernatants, which do not require affinity purification, and with consistent immunoglobulin reactivity.

The aim of this study was to investigate the possible use for routine immunohistopathological diagnosis of a monoclonal anti-human prostatic acid phosphatase monoclonal antibody, PASE/4LJ (Haines *et al.*, 1987), by examining its reactivity towards a wide variety of prostatic and non-prostatic normal and malignant tissues. We also examined whether the antibody had any inhibitory effect on PAP enzyme activity, to determine if it could be of any potential in an immuno-assisted enzyme assay. The antibody was initially produced in preference to an anti-PSA antibody, in order to be able to quantitate and compare immunohistochemical staining with acid phosphatase enzyme histochemistry.

### Materials and methods

Prostatic acid phosphatase was purified by ammonium sulphate precipitation, concanavalin A chromatography and ion exchange chromatography, and a monoclonal antibody to PAP (PASE/4LJ) was produced as previously reported (Haines *et al.*, 1987). Tissue culture supernatant was used for immunostaining. Ascitic fluid was produced in the peritoneal cavity of pristane primed Swiss A2G mice by intraperitoneal injection of $5 \times 10^7$ hybridoma cells. Fluid was removed after 10–14 days, centrifuged and stored at $-20°C$.

Purified PAP (5.6 mg) was coupled to 5 ml Affi-gel 15 according to the method recommended in the manufacturer's

*Now at the Department of Applied Science, Leeds Polytechnic, Leeds, UK.
†Now at the Department of Histopathology, West Middlesex University Hospital, Middlesex, UK.
Correspondence: E. Heyderman.
Received 3 April 1989; and in revised form 8 August 1989.

bulletin 1085 (Bio-Rad Laboratories Ltd, Herts.).

Affinity purified antibody was produced from ascites by applying ascitic fluid diluted 1:1 in PBS to a column of PAP-coupled Affi-gel 15. The column was washed with PBS, and specific antibody eluted with 3 M guanidine hydrochloride. Pooled fractions were dialysed against PBS, and then distilled water, and lyophilised.

*Preparation of negative control antibody*

An absorbed control supernatant was prepared by incubating 1 ml of PASE/4LJ supernatant with 1 ml of PAP-coupled Affi-gel 15 agarose beads. The supernatant was separated from the beads by centrifugation and then used at the same dilution as the positive antibody. As a control for non-specific binding to Affi-gel 15, PASE/4LJ supernatant was similarly incubated with Affi-gel 15 coupled to ovalbumin and also to Affi-gel 15 blocked only with ethanolamine-HCl.

*Test of inhibition of PAP by monoclonal antibody*

Doubling dilutions in 1% ovalbumin in PBS from $0.85\,mg\,ml^{-1}$ to $1.6\,\mu g\,ml^{-1}$ of affinity purified PASE/4LJ, mouse IgG (Sigma Chemical Co. Ltd, Dorset), and an irrelevant mouse monoclonal antibody to human chorionic gonadotropin (beta-HCG; Unipath, Herts.) were each incubated in 96-well microtitre plates with $1\,\mu g\,ml^{-1}$ of purified PAP (total volume $150\,\mu l$) for 2 h at room temperature. Acid phosphatase activity was assayed by a standard method (van Etten & Saini, 1977), except that the assay was carried out in microtitre plates and only $20\,\mu l$ of sample was used, volumes of other reagents being reduced accordingly. Absorbence at 410 nm was measured using a plate reader (Dynatech Laboratories Ltd, Sussex).

*Tissues*

Routinely processed formalin-fixed paraffin-embedded blocks were selected from both male and female patients. These consisted of 32 primary tumours of the prostate, 11 metastatic prostate carcinomas, 12 hyperplastic prostates, 402 benign and malignant tumours of other organs, and 191 blocks of non-neoplastic non-prostatic tissues. In addition, eight animal prostates (from dog, rabbit and rat) were selected.

Cryostat sections of non-neoplastic prostatic chippings were prepared, and chippings from another patient were fixed in unbuffered formalin, phosphate buffered formalin, formal acetic acid, Bouin's, formal sublimate or methacarn, and processed to paraffin wax. Other chippings were fixed in unbuffered formalin and treated with either of two decalcification protocols; 33% (v/v) formic acid in 12.5% (w/v) citric acid for 2 days, or 12.5% (w/v) EDTA for 2 days and then wax embedded.

*Immunocytochemistry*

Sections of $4\,\mu m$ were immunostained using an indirect immunoperoxidase technique (Heyderman *et al.*, 1986), with tissue culture supernatant diluted 1:10 in PBS-OA, and a sheep anti-mouse horseradish peroxidase conjugate (Amersham International plc, Bucks.) diluted 1:40 in PBS-OA. A positive control slide of hyperplastic prostate was included with each group of sections stained. The presence of normal tissue adjacent to tumour was noted and included with the normal tissue results.

Fetal and adult normal kidneys were also stained by the streptavidin-biotin ABC method, using PASE/4LJ diluted 1:100 in PBS-OA, and biotinylated horseradish peroxidase and streptavidin (Hsu *et al.*, 1981) (Dako Ltd, Bucks.). This dilution of antibody was found to produce strong positive staining in the control slide of prostate. Staining of the kidneys was then repeated at a dilution of 1:10.

Sequential sections of each positive block were restained with PASE/4LJ, and with the negative absorbed control reagent. A control block of hyperplastic prostate was also stained with the PASE antibody incubated either with Affi-Gel 15 coupled to mouse immunoglobulin, or to ovalbumin.

A Perls stain (Stevens, 1982) was carried out to confirm the presence of iron on the sections in which a greenish brown pigment was present. Sections containing an unidentified brownish pigment in negative controls, interpreted as probably being lipofuscin, were stained by the Schmorl method (Stevens, 1982).

The antibody was also used for 6 months in the routine immunocytochemistry laboratory in parallel with an affinity purified rabbit polyclonal antibody to PAP (Heyderman *et al.*, 1984). The same indirect technique was used.

### Results

*Inhibition of PAP*

PASE/4LJ had no effect on the enzyme activity of PAP; neither did mouse IgG or the irrelevant monoclonal antibody to beta-HCG at up to 800-fold molar excess of immunoglobulin over PAP.

*Tissue staining*

A total of 658 blocks of tissues were stained with the monoclonal antibody, PASE/4LJ (see Tables I, II and III). All 12 benign, 32 malignant, 11 metastatic and two fetal prostates were positive (Figures 1, 2 and 3). There were always some negative areas of epithelium, and the intensity of staining varied, generally being weaker in carcinomas than in hyperplastic prostates. Staining was seen in scattered cells within the urothelium of adult, fetal prostatic and penile urethra (5/6) (Figure 4). There was a variable degree of staining of prostatic fibromuscular stroma, though smooth muscle elsewhere was negative.

Nine of 402 non-prostatic neoplasms were positive. The staining in these tissues varied in intensity, but in none was it as strong as in prostatic epithelium. Six of 36 carcinoids, 1/55 ovarian adenocarcinomas (a serous adenocarcinoma), 1/1 carcinosarcoma of the lung (adenocarcinomatous foci) and 1/9 islet cell tumours were positive. There was staining of islet cells in 4/5 blocks of normal pancreas (Figure 6) and in 4/9 blocks of non-neoplastic pancreas surrounding tumour. In addition, the loops of Henle, maculae densae and distal tubules in 10/10 fetal kidneys (Figure 5) and 2/9 adult kidneys were positive, while proximal tubules and collecting ducts were negative. Three of 10 fetal kidneys and 1/2 positive adult kidneys were from females. Staining of fetal kidney was stronger than that in adult kidney.

The fetal and adult kidneys and positive hyperplastic control section were also positive when the ABC method was used with the antibody diluted 1:100. No additional adult kidneys were positive even when the primary antibody was used at a dilution of 1:10, i.e. at 10 times the concentration required for strong staining of the control hyperplastic prostate.

**Table I** Tissues positively stained with monoclonal antibody to prostatic phosphatase PASE/4LJ

| |
|---|
| All normal and hyperplastic prostatectomy specimens (12) plus non-neoplastic prostatic glands included in bladder biopsies, all primary adenocarcinomas of the prostate (32), scattered cells in prostatic, bulbar and penile urethra (5/6), prostatic carcinoma invading rectum (1), prostatic metastase to lymph nodes (5) and bone (5). |
| 1/55 ovarian carcinomas (1/29 serous, 0/5 mucinous, 0/6 clear cell, 0/9 endometrioid, 0/6 poorly differentiated). |
| 6/36 carcinoids (1/2 colorectal, 1/7 appendix, 4/12 small bowel, 0/3 stomach, 0/11 lung, 0/1 testis). |
| 1/9 pancreatic islet cell tumours (an islet cell carcinoma). |
| Pancreatic islets – cells stained in some islets (4/5 blocks of normal pancreas, 4/9 blocks of uninvolved pancreas surrounding pancreatic tumour). |
| Kidney – staining of loops of Henle, maculae densae, and distal tubules of 10/10 fetal and 2/9 adult kidneys. |

**Table II** Tumours negative for PAP (total 393/402)

Urinary tract (20) – transitional cell carcinoma of the bladder (12), adenocarcinoma of the bladder (2), transitional cell carcinoma of the ureter (1), transitional cell carcinoma of the prostate (1), hypernephroma (5), nephroblastoma (1).

Genital tract (78) – testicular teratoma (4), testicular teratoma plus seminoma (1), seminoma (3), testicular yolk sac tumour (1), carcinoma of the cervix (5), endometrial adenocarcimona (4), ovarian choriocarcinoma (1), dysgerminoma (1), ovarian yolk sac tumour (2), fibroids (2), ovarian adenocarcinomas: serous (28 negative, 1 positive), mucinous (5), clear cell (6), endometroioid (9), poorly differentiated (6).

Endocrine organs (62) – adrenal carcinoma (5), adrenal cortical adenoma (7), phaeochromocytoma (6), islet cell adenoma (3), islet cell carcinoma (5 negative, 1 positive), pituitary adenoma (4), thyroid adenoma (1), parathyroid adenoma (6), carcinomas of the thyroid: anaplastic (5), follicular (7), papillary (8), medullary (5).

Carcinoids (30) – appendix (6 negative, 1 positive), colorectal (1 negative, 1 positive), ileum (8 negative, 4 positive), lung (11), stomach (3), testis (1).

Bone, muscle and soft tissue (38) – chondrosarcoma (4), chordoma (2), exostosis and osteoid osteoma (3), non-ossifying fibroma (4), osteoclastoma (6), osteosarcoma (2), dermatofibrosarcoma protuberans (1), epitheloid sarcoma (92), liposarcoma (4), malignant fibrous histiocytoma (3), malignant nerve sheath tumour (2), rhabdomyosarcoma (3), synovial sarcoma (2).

Breast (32) – fibroadenoma (5), ductal carcinoma (10 female and 2 male), lobular (9), medullary (2), cystosarcoma phylloides (4).

Gastrointestinal tract (44) – colorectal carcinoma (12), gastric carcinoma (7), pancreatic adenocarcinoma (8), hepatocellular carcinoma (3), hepatoblastoma (2), salivary gland tumours: adenocarcinoma (3), adenocystic carcinoma (4), mucoepidermoid carcinoma (2), acinic cell tumour (2), pleomorphic adenoma (1).

Respiratory system (36) – lung adenocarcinoma (17), lung squamous carcinoma (2), lung large cell anaplastic carcinoma (4), lung oat cell carcinoma (2), bronchio-alveolar carcinoma (1), mesothelioma (10).

Lympho-proliferative system (17) – lymphoma non-Hodgkin's (8), Hodgkin's (5), chloroma (2), thymoma (2).

Skin (17) – basal cell carcinoma (3), squamous carcinoma (3), sebaceous carcinoma (3), malignant eccrine poroma (3), Merkel cell tumour (2), secondary melanoma in nodes (2).

Nervous system (18) – astrocytoma (6), medulloblastoma (3), ependymona (2), choroid plexus tumour (1), craniopharyngioma (1), meningioma (5).

**Table III** Normal and non-neoplastic tissues negative for prostatic acid phosphatase

*Adult tissues*

Either part of a larger resection specimen for a non-malignant condition, from uninvolved tissue surrounding tumour, or from autopsy material.

Genitourinary system – kidney, ureter, bladder, ovary, testis, endometrium (proliferative, secretory, 'pill' type, atrophic), myometrium, breast (normal, lactating, fibrocystic disease, gynaecomastia).

Lympho-proliferative system – tonsil, thymus, lymph nodes, spleen, bone marrow.

Musculoskeletal and nervous system – striated and smooth muscle, bone, cartilage.

Cardiovascular and respiratory system – veins, arteries, cardiac muscle, lung parenchyma, bronchus, pleura.

Endocrine and glandular tissue – pituitary, thyroid, adrenal, parathyroid, salivary glands.

Gastrointestinal tract – oesophagus, stomach, small and large bowel including rectum.

Nervous system – cerebrum, cerebellum, spinal cord, eye.

*Fetal tissues*

Lung, liver, spleen, ovary, testis, adrenal, bladder, heart, gallbladder, pancreas, thymus, thyroid, brain, first, second and third trimester placenta, umbilical cord.

*Animal tissues*

Prostates from dog, rabbit and rat.

Staining of all of the previously positive sections was repeated and in parallel with the control absorbed antibody absorbed with PAP coupled Affi-gel 15. All remained positive with PASE/4LJ, and were negative with the control antibody, although there was iron pigment in some sections and unidentified pigment, probably lipofuscin, in others, present in both test and control sections. Incubation of the monoclonal antibody with Affi-gel 15, either coupled to ovalbumin or with the active sites blocked with ethanolamine, did not reduce the intensity of staining of the positive control hyperplastic prostate, confirming the specificity of the absorption procedure.

Staining was observed in keratinised cells in skin sections. This staining was not present when the absorbed control antibody was used. However, it was not regarded as specific since it has been seen when other monoclonal antibodies such as anti-CEA were used (Haines *et al.*, unpublished data). This may represent either non-specific binding of mouse Ig, or binding of IgG to Fc receptors still present in fixed tissues (Garvin *et al.*, 1974).

Hyperplastic prostatic chippings, frozen and unfixed, fixed in each of the seven different fixatives, or treated with either of the two decalcification protocols, stained positively, as did bone trephines from deposits of metastatic prostatic tumour decalcified in EDTA or in formic/citric acid. The antibody did not stain any of the eight animal prostates (four dog, three rat, one rabbit); neither did it stain sections of one of the canine prostates fixed in each of seven fixatives.

Unidentified pigment was seen in some endocrine cells, particularly the parathyroids, and in one islet cell tumour, and it was present in both the test and the negative control slides. It stained positively with a Schmorl stain, and was interpreted as possibly lipofuscin, but not identified further. Perls stains confirmed the presence of haemosiderin in areas considered to contain iron in immunostained preparations.

PASE/4LJ produced comparable staining to the rabbit anti-PAP antibody in all the tissues tested for PAP in the routine immunocytochemistry laboratory.

### Discussion

This report describes a murine monoclonal antibody to prostatic acid phosphatase, PASE/4LJ, which can be used to immunostain frozen sections, paraffin-embedded tissue fixed in a variety of fixatives, and decalcified blocks. All of the primary and metastatic prostatic carcinomas were positive, and gastrointestinal and transitional cell carcinomas were negative, indicating that the absence of PAP would make the diagnosis of a prostatic primary or secondary most unlikely. However, positivity was seen in some non-prostatic malignant and normal tissues.

The presence of PAP positive cells in the penile and prostatic urethra would explain the occurrence of 'ectopic' prostatic tissue in these sites. Haematuria and dysuria have been associated with their presence (Heyderman *et al.*, 1987).

The antibody shares the previously described recognition of an epitope(s) in some carcinoids of the gastrointestinal tract (Sobin *et al.*, 1986), pancreatic islet cells, and islet cell tumours (Choe *et al.*, 1978; Jobsis *et al.*, 1981; Yam *et al.*, 1981; Cohen *et al.*, 1983). The proportion of gastrointestinal carcinoids positive for PAP appears to be maximal distally (Sobin *et al.*, 1986), and the positivity of rectal carcinoids may be a problem if the antibody is to be used for the differential diagnosis of tumours involving the wall of the rectum with no apparent mucosal origin. Positive staining for both prostatic acid phosphatase and prostate specific antigen have been reported in 'carcinoid-like' tumours of the prostate which are argyrophilic, but lack other markers of neurendocrine differentiation (Ansari *et al.*, 1981; Almagro *et al.*, 1986). Dispute as to whether or not these should be considered 'true' carcinoids remains.

Staining was seen in the loops of Henle, maculae densae and distal tubules of all 10 fetal kidneys and in 2/9 adult kidneys. The staining could not be detected in the seven negative adult normal kidneys even when the ABC method was used at a 10 times higher concentration of PASE/4LJ normally used for this method. The staining was not sex-specific, since 3/10 fetal kidneys and 1/2 positive adult kidneys were from females. The antibody was therefore unlikely to be demonstrating material derived from the prostate, and absorbed by the renal tubules. It may represent recognition of a renal acid phosphatase or an epitope on an

**Figure 1** Specimen of benign prostatic hypertrophy showing good discrimination of staining between epithelial cells and stroma. In some specimens, staining of prostatic fibromuscular stroma was more marked. × 45.

**Figure 2** Residual benign prostatic glands encircled by well differentiated prostatic adenocarcinoma. In this field there is patchy staining of non-neoplastic epithelium, with more uniform staining of the tumour. In most other specimens, benign glands were more intensely stained than tumour. × 45.

**Figure 3** Decalcified bone trephine through deposit of osteosclerotic metastatic prostatic carcinoma. The tumour is positive; osteoblasts rimming the bone trabeculae are negative. × 75.

**Figure 4** Penile urethra showing focal differentiation of epithelium into prostatic acid phosphatase secreting cells. There is surrounding fibrosis in this specimen with some distortion of the lumen. × 45.

**Figure 5** Fetal kidney (female) with glomeruli negative, and loops of Henle positive. × 75.

**Figure 6** Section of normal pancreas showing staining of some cells in all of the islets. × 75.

unrelated molecule. Since both intensity and occurrence of staining were less in adult than fetal kidneys, the epitope could be related to differentiation. A study of fetal kidneys at various gestation ages would be required to investigate this further.

Staining of some renal tubules has also been reported with monoclonal antibodies raised against antigens other than PAP or PSA (Frankel *et al*., 1982*a*), as well as with some monoclonal anti-PSA antibodies (Frankel *et al*., 1982*b*), and polyclonal PAP antisera (Yam *et al*., 1981). Frankel *et al*. explained staining of the kidney as being due to non-idiotypic binding of the mouse immunoglobulin to a receptor(s) on tubule epithelium. However, staining of similar tissues with an anti-EMA monoclonal antibody (Heyderman *et al*., 1985) showed a different pattern of distribution, with distal and collecting tubules positive (Cordell *et al*., 1985). Localisation in specific areas of the renal tubules may be of diagnostic use for differentiating between the various regions of the nephron in damaged kidneys, when the distal tubules may be difficult to distinguish from the vasa recta.

One ovarian adenocarcinoma and one pulmonary carcinosarcoma were positive, but positivity in an ovarian carcinoma is not likely to give rise to diagnostic problems. Although 26 epithelial lung tumours were negative, the carcinosarcoma showed weak positivity in the adenocarcinomatous portion. It would be most unusual for a prostatic carcinoma to present initially with a lung deposit and no evidence of a prostatic primary, but the antibody might be of limited value in the differential diagnosis of primary adenocarcinoma of the lung from a prostatic metastasis. However, staining in these two tumours was extremely weak, unlike that seen in tissues of prostatic origin (Figures 1–3).

The colorectal, transitional cell carcinomas and bladder adenocarcinomas were all negative with PASE/4LJ monoclonal antibody, indicating its value in the differential diagnosis of tumours arising in rectum, prostate and bladder. In general, transitional carcinomas of the bladder may be identified by their characteristics morphology, but glandular metaplasia and/or diathermy artefact may make the diagnosis difficult, especially around the bladder neck.

The antibody did not stain canine prostates, although staining of canine prostates with anti-human PAP antibodies has been reported (McEntee *et al*., 1987), nor did it stain those of rat or rabbit. However, the number studied was small and a much larger study might well reveal activity in some instances.

Staining of a variety of other tissues has been noted in previous reports using polyclonal antisera to PAP. With the PASE/4LJ monoclonal antibody no staining was seen in granulocytes (Yam *et al*., 1981), osteoclasts (Jobsis *et al*., 1981), parietal cells of the stomach, liver cells, renal cell or breast carcinomas (Li *et al*., 1980; Yam *et al*., 1981).

Antisera to prostate specific antigen may be superior for monitoring of disease progression by serum RIA (Stamey *et al*., 1987). In some reports more prostatic tumours appear to stain positively for PAP than for PSA (Vernon & Williams, 1983; Keillor & Aterman, 1987), while in others more were positive for PSA (Allhoff *et al*., 1983; Shah *et al*., 1985). These different results may well reflect differences in the antibodies used for immunostaining.

PASE/4LJ does not inhibit the enzyme activity of PAP and is therefore unlikely to bind to the active site. This indicates that the antibody could be of potential use in an immuno-assisted enzyme assay for PAP in serum.

In conclusion, the antibody has proved a useful addition to our diagnostic histopathology armamentarium, being used successfully in this department for the differential diagnosis of primary prostatic, bladder and rectal carcinomas, and for the investigation of metastatic deposits of tumours of unknown origin.

This study would not have been possible without the support and encouragement of Sir Denis Hamilton and Dr David Collins. We should like to thank our colleagues for providing blocks or sections of tissues difficult for us to obtain, and the technical staff of the Department of Histopathology for cutting many of the sections required for this work. The monoclonal anti-HCG beta was a gift from Unipath Ltd. This work was supported by the Nutbourne Trust, the Wolfson Foundation, St Thomas Hospital Research and Endowments Fund, and a National Westminster Bank grant for Cancer Research. PASE/4LJ is available from DAKO Ltd, High Wycombe, Bucks., to whom all enquiries for the antibody should be directed.

### References

ABDUL-FADL, M.A.M. & KING, E.J. (1949). Properties of the acid phosphatases of erythrocytes and of the human prostate gland. *Biochem. J.*, **45**, 51.

ALLHOFF, E.P., PROPPE, K.H., CHAPMAN, C.M., LIN, C.-W. & PROUT, G.R. (1983). Evaluation of prostate specific acid phosphatase and prostate specific antigen in identification of prostatic cancer. *J. Urol.*, **129**, 315.

ALMAGRO, U.A., TIEU, T.M., REMENIUK, E., KUECK, B. & STRUMPF, K. (1986). Argyrophilic, 'carcinoid-like' prostatic carcinoma. An immunocytochemical study. *Arch. Pathol. Lab. Med.*, **110**, 916.

ANSARI, M.A., PINTOZZI, R.L., CHOI, Y.S. & LADOVE, R.F. (1981). Diagnosis of a carcinoid-like metastatic prostatic carcinoma by an immunoperoxidase method. *Am. J. Clin. Pathol.*, **76**, 94.

BARKA, T. (1960). A simple azo method for histochemical demonstration of acid phosphatase. *Nature*, **187**, 248.

BURNS, J. (1977). Prostatic acid phosphatase in tissue sections revealed by the unlabelled antibody peroxidase-antiperoxidase method. *Biomedicine*, **27**, 7.

CHOE, B.K., PONTES, E.J., ROSE, N.R. & HENDERSON, M.D. (1978). Expression of human prostatic acid phosphatase in a pancreatic islet cell carcinoma. *Invest. Urol.*, **15**, 312.

COHEN, C., BENTZ, M.S. & BUDGEON, L.R. (1983). Prostatic acid phosphatase in carcinoid and islet cell tumors. *Arch. Pathol. Lab. Med.*, **107**, 277.

CORDELL, J., RICHARDSON, T.C., PULFORD, K.A.F. & 4 others (1985). Production of monoclonal antibodies against human epithelial membrane antigen for use in diagnostic immunocytochemistry. *Br. J. Cancer*, **52**, 347.

FRANKEL, A.E., ROUSE, R.V. & HERZENBERG, L.A. (1982*a*). Human prostate specific and shared differentiation antigens defined by monoclonal antibodies. *Proc. Natl Acad. Sci. USA*, **79**, 903.

FRANKEL, A.E., ROUSE, R.V., WANG, M.C., CHU, T.M. & HERZENBERG, L.A. (1982*b*). Monoclonal antibodies to a human prostate antigen. *Cancer Res.*, **42**, 3714.

GARVIN, A.J., SPICER, S.S., PARMLEY, R.T. & MUNSTER, A.M. (1974). Immunohistochemical demonstration of IgG in Reed-Sternberg and other cells in Hodgkin's disease. *J. Exp. Med.*, **139**, 1077.

GIESELMANN, V., HASILIK, A. & VON FIGURA, K. (1984). Tartrate-inhibitable acid phosphatase. Purification from placenta, characterisation and subcellular distribution in fibroblasts. *Hoppe-Seyler's Z. Physiol. Chem.*, **365**, 651.

HAINES, A.M.R., LARKIN, S.E. & HEYDERMAN, E. (1987). A new monoclonal antibody to human prostatic acid phosphatase suitable for immunohistology in formalin-fixed paraffin-embedded tissue sections. *Biochem. Soc. Trans.*, **15**, 1179.

HEYDERMAN, E., BROWN, B.M.E. & RICHARDSON, T.C. (1984). Epithelial markers in prostatic, bladder, and colorectal cancer: an immunoperoxidase study of epithelial membrane antigen, carcinoembryonic antigen, and prostatic ad phosphatase. *J. Clin. Pathol.*, **37**, 1363.

HEYDERMAN, E., MANDALIYA, K.N., O'DONNELL, P.J., KADOW, C. & BULTITUDE, M.I. (1987). Ectopic prostatic glands in the bulbar urethra. An immunoperoxidase study. *Urology*, **29**, 76.

HEYDERMAN, E. (1986). Tumour markers. In *Immunocytochemistry: Modern Methods and Applications, 2nd Edn*, Polak, J.M. & Van Noordan, S. (eds) p. 502. John Wright: Bristol.

HEYDERMAN, E., STRUDLEY, I., POWELL, G., RICHARDSON, T.C., CORDELL, J.L. & MASON, D.Y. (1985). A new monoclonal antibody to epithelial membrane antigen (EMA)-E29. A comparison of its immunocytochemical reactivity with polyclonal anti-EMA antibodies and with another monclonal antibody, HMFG-2. *Br. J. Cancer*, **52**, 355.

HSU, S.-M., RAINE, L. & FANGER, H. (1981). Use of avidin-biotin-peroxidase complex (ABC) in immunoperoxidase techniques: a comparison between ABC and unlabeled antibody (PAP) procedures. *J. Histochem. Cytochem.*, **29**, 577.

JOBSIS, A.C., DE VREIES, G.P., ANHOLT, R.R.H. & SANDER, G.T.B. (1978). Demonstration of the prostatic origin of metastases. An immunocytochemical method for formalin-fixed embedded tissue. *Cancer*, **41**, 1788.

JOBSIS, A.C., DE VREIES, G.P., MEIJER, A.E.F.H. & PLOEM, J.S. (1981). The immunohistochemical detection of prostatic acid phosphatase: its possibilities and limitations in tumour histochemistry. *Histochem. J.*, **13**, 961.

KEILLOR, J.S. & ATERMAN, K. (1987). The response of poorly differentiated prostatic tumours to staining for prostate specific antigen and prostatic acid phosphatase: a comparative study. *J. Urol.*, **137**, 894.

KOHLER, G. & MILSTEIN, C. (1975). Continuous cultures of fused cells secreting antibody of predefined specificity. *Nature*, **256**, 495.

LI, C.-Y., LAM, W.K.W. & YAM, L.T. (1980). Immunohistochemical diagnosis of prostate cancer with metastasis. *Cancer*, **46**, 706.

LI, C.Y., YAM, L.T. & LAM, K.W. (1970). Studies of acid phosphate isoenzymes in human leukocytes: demonstration of isoenzyme cell specificity. *J. Histochem. Cytochem.*, **18**, 901.

LUNQUIST, F. (1946). Function of prostatic acid phosphatase. *Nature*, **158**, 710.

MANN, T. (1964). *The Biochemistry of Semen and of the Male Reproductive Tract*. John Wiley & Sons: Chichester.

MCENTEE, M., ISAACS, W. & SMITH, C. (1987). Adenocarcinoma of the canine prostate: immunohistochemical examination for secretory antigens. *Prostate*, **11**, 163,

OFFICE OF POPULATION CENSUS AND SURVEYS (1988*a*). *Cancer Statistics: Registrations 1984 Series MB1 (16)*, p. 4. HMSO: London.

OFFICE OF POPULATION CENSUS AND SURVEYS (1988*b*). *Mortality Statistics: Cause 1986 Series DH2 (13)*, p. 8. HMSO: London.,

SAINI, M.S. & VAN ETTEN, R.L. (1978). A homogenous isoenzyme of human liver acid phosphatase. *Arch. Biochem. Biophys.*, **191**, 613.,

SHAH, N.T., TUTTLE, S.E., STROBEL, S.L. & GANDHI, L. (1985). Prostatic carcinoma metastatic to bone: sensitivity and specificity of prostate-specific antigen and prostatic acid phosphatase in decalcified material. *J. Surg. Oncol.*, **29**, 265.

SOBIN, L.H., HJERMSTAD, B.M., SESTERHENN, I.A., & HELWIG, E.B. (1986). Prostatic acid phosphatase activity in carcinoid tumours. *Cancer*, **58**, 136.

STAMEY, T.A., YANG, N., HAY, A.R., MCNEAL, J.E., FREIHA, F.S. & REDWINE, E. (1987). Prostate-specific antigen as a serum marker for adenocarcinoma of the prostate. *N. Engl. J. Med.*, **317**, 909.

STEVENS, A. (1982). Pigments and minerals. In *Theory and Practice of Histological Techniques*, 2nd edn, Bancroft, J.D. & Stevens, A. (eds) p. 242. Churchill Livingstone: Edinburgh.

VAN ETTEN, R.L. & SAINI, M.S. (1977). Preparation of homogenous human prostatic acid phosphatase using concanavalin A-sepharose 4-B. *Biochim. Biophys. Acta*, **484**, 487.

VERNON, S.E. & WILLIAMS, W.D. (1983). Pre-treatment and post-treatment evaluation of prostatic adenocarcinoma for prostatic specific acid phosphatase and prostatic specific antigen by immunohistochemistry. *J. Urol.*, **130**, 95.

WAHEED, A., VAN ETTEN, R.L., GIESELMANN, V. & VON FIGURA, K. (1985). Immunological characterisation of human acid phosphatase gene products. *Biochem. Genet.*, **23**, 309.

WANG, M.C., VALENZUELA, L.A., MURPHY, G.P. & CHU, T.M. (1979). Purification of a human prostate specific antigen. *Invest. Urol.*, **17**, 159.

WATT,. K.W.K., LEE, P.-J., M'TIMKULU, T., CHAN, W.-P. & LOOR, R. (1986). Human prostate-specific antigen: structural and functional similarity with serine proteases. *Proc. Natl Acad. Sci. USA*, **83**, 3166.

WOLJCIESZYN, J.W., WANG, M.C, LEE, C.L., MURPHY, G.P. & CHU, T.M. (1979). Purification and characterisation of a human urinary acid phosphatase. *J. Appl. Biochem.*, **1**, 223.

YAM, L.T., JANCKILA, A.J., LAM, W.K.W. & LI, C.-Y. (1981). Immunohistochemistry of prostatic acid phosphatase. *Prostate*, **2**, 97.

ZWIRNER, A., SEITZ, J. & AUMULLER, G. (1986). Biochemical and immunological relationships of prostatic and leukocytic acid phosphatases and their subcellular localisation. *Acta Biochim. Pol.*, **33**, 125.